# Effect of AlOx protection layer on AgNWs for flexible transparent heater

**DOI:** 10.1038/s41598-020-61449-6

**Published:** 2020-03-12

**Authors:** Joon-Min Lee, Young-Hoi Kim, Han-Ki Kim, Hye-Jin Kim, Chan-Hwa Hong

**Affiliations:** 1 Electronics and Telecommunications Research Institute, 218, Gajeong-ro, Yuseong-gu, Daejeon South Korea; 2 University of Science and Technology, 217, Gajeong-ro, Yuseong-gu, Daejeon South Korea; 3 School of Advanced Materials Science & Engineering, Sungkyunkwan University, Gyeonggi-do, 440-746 South Korea

**Keywords:** Materials for devices, Ceramics, Other nanotechnology

## Abstract

We indicated high performance and stability transparent heaters based on AlOx covered Ag nanowires. We obtained an AlOx covered Ag nanowire thin film which has a 47 ohm/sq of sheet resistance and 88.1% (substrate included) of transmittance at 600 nm on a flexible substrate. We demonstrate that the thin AlOx layer leads to increased contact area at the junction of Ag nanowires, which contributes to lower sheet resistance and improved adhesion of Ag nanowires. Furthermore, high stability and flexibility of Ag nanowire have been achieved by the AlOx layer. Finally, we fabricated a flexible transparent heater with AlOx covered Ag nanowire, and obtained a temperature of 81 °C within 40 sec at the driven voltage of 7 V with fast response and uniform temperature distribution. Therefore, the AlOx covered Ag nanowire film is a promising candidate for the application of the flexible transparent heaters.

## Introduction

Transparent heaters are commonly used in a wide range of applications such as solar panels, vehicle defrosters, periscopes, and smart, heat-retaining windows. Especially, the future direction of flexible transparent heaters focuses on anti-fogging windshields, mirrors, and displays ensuring the fast response of electronic devices under cold and icy environmental conditions^1–4^. For a high-performance transparent heater, a high quality transparent conductive material is essential. In order to meet the requirement of high conductivity with transparency and flexibility, carbon-based materials (graphene, carbon nanotubes (CNTs))^5–11^, conducting polymers^12–15^, metal nanoparticles^16^, and metal mesh^17–19^ have been widely used for a flexible substrate. Although the flexibility of these transparent conductors is greatly improved, their performance highly depends on the sample preparation and often does not meet the requirement for many applications regarding conductivity and stability^20–22^. However, metal nanowires (NWs) in the form of randomly percolation network have shown excellent potential as flexible transparent conductors^23–29^. Thus, metal nanowires with low resistance, high transmittance and stability are very important parts for future applications. Especially, AgNW-based applications have been actively researched because of its exceptional properties: LED array, touch-panels, displays, anti-counterfeit devices, energy harvester, skin attachable and implantable sensors, flexible and stretchable transparent heaters^30–36^. However, in the case of a metal nanowire, many researchers have realized the serious problem of metal corrosion^37,38^. Furthermore, the poor adhesion of metal network to the substrate limits its wide applications^23,39^, high stability against humidity and period of metal nanowires have not yet been achieved simultaneously for transparent conductive materials. Hwang *et al*. reported that a 5.3 nm thickness Al_2_O_3_ using atomic layer deposition (ALD) method can improve the thermal and mechanical stability of Ag nanowire electrodes^40^. However, ALD is not suitable for mass production because of a time-consuming process and toxic chemicals. And its higher temperature process over 100 °C limits a variety of flexible substrates. To overcome these problems, we investigated the effect of the AlOx protection layer on Ag nanowires using physical vapor deposition (PVD) method to gain a good adhesion and stability with high transmittance and conductivity. The whole process was carried out at room temperature without any annealing process and chemical treatment. Comparison with Ag nanowires, electrical properties and adhesion of AlOx covered Ag nanowires were improved, AlOx covered Ag nanowires can be obtained without serious conductivity and transmittance loss. Finally, we demonstrate the fabrication of a highly transparent, conductive, and stable AlOx covered Ag nanowires based on a flexible transparent heater, 81 °C of temperature within 40 sec at the driven voltage of 7 V has been achieved. These results are a promising application for the flexible transparent heater. The results show that the AlOx protection layer on Ag nanowires can significantly reduce the sheet resistance and increased stability of Ag nanowires, which can enhance heat generation behavior and duration of usage for flexible transparent heater applications.

## Experiments

AgNWs were coated on a 100 *μ*m-thick Polyethylene terephthalate (PET) substrate by a drop-casting method. Then, the coated substrate was dried for 30 sec in the air wind with a temperature of 60–65 °C and a velocity of 17 m/s^41^. The typical diameter of AgNWs (NANOPYXIS Inc.) was 30 nm and their length was 5~10 μm. The AgNWs were dispersed in Isopropanol (IPA) with a concentration of 0.15 wt%. After we made the AgNWs on the PET substrate, we deposited AlOx with a radio frequency (RF) power of 700 W in the vacuum chamber with the 3.5 mTorr of working pressure. Al target (99.99%) was used for the deposition, Ar-diluted O2 gas (5%) was used for reactive sputtering deposition. The sheet resistance of the AgNWs was measured using a four-point probe. Surface morphology images of the samples were obtained using a field-emission scanning electron microscopy (FE-SEM). The optical transmittance was measured in the wavelength range of 400–1000 nm by UV-spectrophotometer. The material components of the thin films were analyzed by using energy dispersive spectrometry (EDS) and X-ray photoelectron spectroscopy (XPS). The mechanical adhesive force was tested using 3 M SCOTCH MAGIC tape, No. 810 (adhesion strength to steel, 2.737 N/cm)^42^. To explain reliability for moisture, the AgNWs films were stored in a thermos-hydrostatic chamber (WEISS WK11 340) for 120 h with a temperature at 85 °C and relative humidity 85%. The voltage source was connected to both edges of the aluminum foil electrode. The temperature and the thermal image of the surface were obtained by a thermal imaging camera (FLIR, TI480).

## Results and Discussion

**Figure 1:**
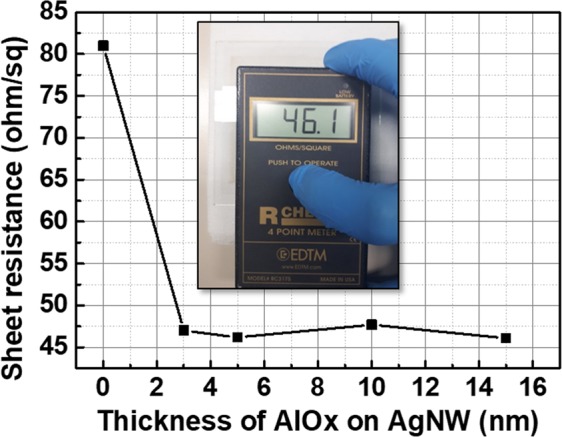
Sheet resistance of AgNWs as a function of AlOx layer thickness.

Figure 1 shows the variation of the sheet resistance of AgNWs as a function of AlOx thickness. As shown in Fig. 1, the sheet resistance of the AgNWs decreased after 3 nm-thick AlOx deposition from 81 ohm/sq to 47 ohm/sq. Then, regardless of AlOx thickness, the sheet resistance of AlOx covered AgNWs were almost the same with 3 nm-thick AlOx covered AgNWs. These results indicate that AlOx thicknesses (3~15 nm) are not related to the sheet resistance of AgNWs.

**Figure 2:**
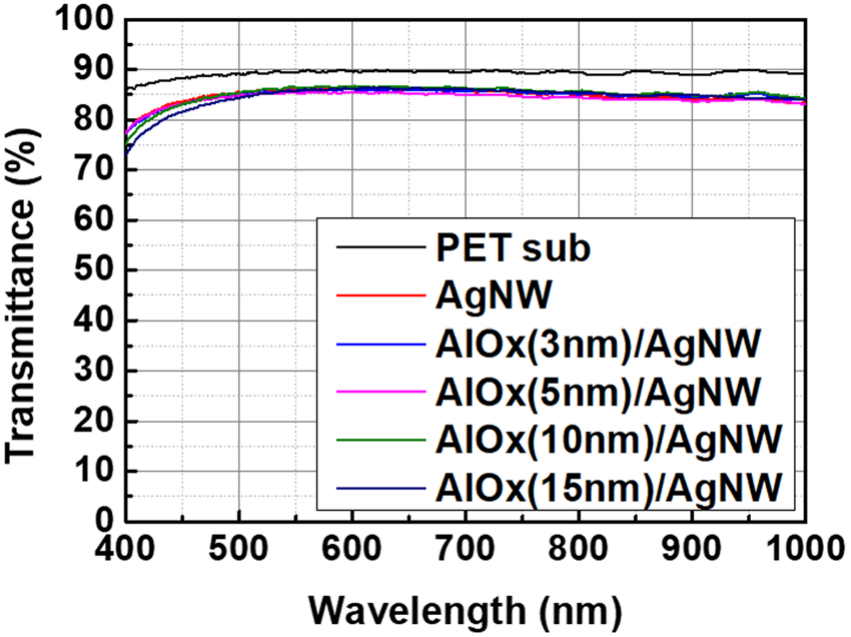
Transmittance of AgNWs as a function of AlOx layer thickness.

Figure 2 shows the change of transmittance of the AgNWs/PET substrate with various AlOx thickness. As shown in Fig. 2, the transmittance (in the whole visible range) of the AgNWs/PET substrate did not change after AlOx deposition. Although the sheet resistance of AgNWs dramatically decreased after AlOx deposition, transmittances did not decrease much after AlOx deposition. Moreover, AgNWs indicate high transparency in the whole visible and infrared range as shown in Fig. 2. These results mean that the AlOx layer does not negatively affect an optical property because AlOx is very transparent. Lots of papers reported that Ag nanowire has a trade-off relationship between transmittance and sheet resistance^43,44^. However, we indicate improved electrical properties of AgNWs using the AlOx layer without transmittance loss.

**Figure 3:**
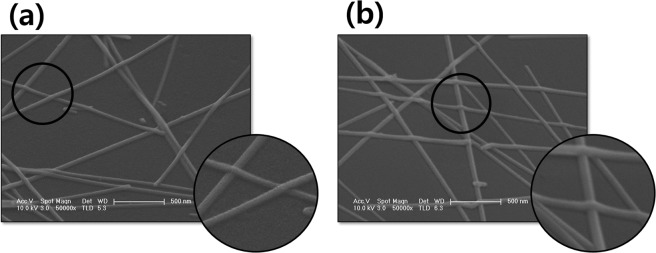
SEM image of AgNWs without AlOx layer (**a**) and with 3 nm-thick AlOx layer (**b**).

To demonstrate the improved electrical properties without the reduction of transmittance after AlOx deposition on the AgNWs, we analyzed surface morphology using an FE-SEM as shown in Fig. 3. Figure 3(a) shows the AgNWs film on the PET substrate. The AgNWs dispersed randomly on a PET substrate, uniformly interconnected AgNWs were formed on a substrate. The contact area at the junction of AgNWs is the dominant factor determining the sheet resistance of AgNWs films^45^. The contact area of AgNW junction parts is small as shown in Fig. 3(a), this result leads to low electrical conductivity. Figure 3(b) shows the SEM image of 3 nm-thick AlOx on dispersed AgNWs. We indicate that after the deposition of AlOx on AgNWs, the contact area of AgNWs junction parts was increased. We expect that plasma energy and a thin film deposition using PVD method reduced the stiffness of AgNWs, the phenomenon leads to increased the contact area of the AgNWs junction^41,46^. As expected from SEM results, the increased contact area of AgNWs junction parts leads to a decrease in the sheet resistance of AgNWs without a reduction of transmittance. EDS measurements were carried out to evaluate the chemical composition of the AlOx thin film.

**Figure 4:**
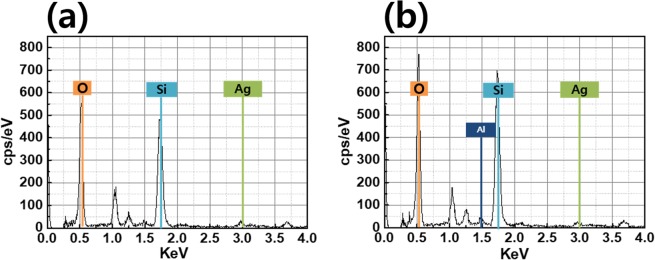
EDX data of AgNWs without AlOx layer (**a**) and with 3 nm-thick AlOx layer (**b**).

As shown in Fig. 4, EDS confirms that AgNWs and AlOx covered AgNWs contains Ag, O, Si peak and Al, Ag, O, Si peak, respectively.

**Figure 5:**
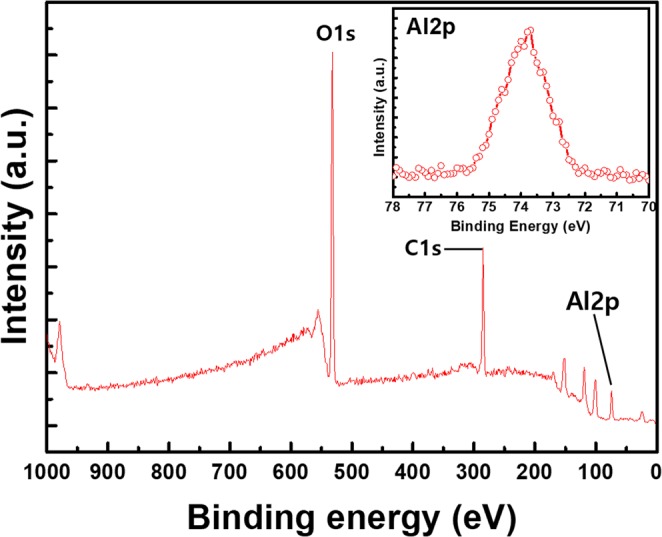
XPS data of 3 nm-thick AlOx covered AgNWs.

To demonstrate the stoichiometry of the AlOx layer, 3 nm-thick AlOx was analyzed using XPS. As a result, the XPS survey spectrum is presented in Fig. 5. The XPS survey spectra of 3 nm-thick AlOx represent mainly Al, O, and C contributions. The binding energy peak of 74 eV indicates Al2p and O1s show ~531 eV peak. Thus, we investigated that Al_2_O_3_ thin film has been deposited by reactive sputtering. We confirmed that the Al_2_O_3_ dominantly existed in the AlOx protection layer.

**Figure 6:**
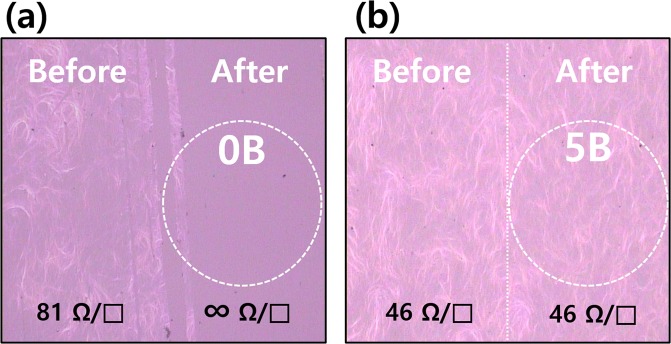
Microscope image (X100) of AgNWs film with 3 nm-thick AlOx (**a**) and without AlOx layer (**b**).

To compare the adhesion of AgNWs with and without the AlOx protection layer, a tape test was carried out as shown in Fig. 6. As shown in Fig. 6(a), the AgNWs without the AlOx layer was easily detached from the PET substrate because of the weak binding energy of the AgNWs on the flexible substrate, and the sheet resistance of detached parts highly increased (∞ ohm/sq). In contrast, the AlOx covered AgNWs exhibited an improvement of adhesion due to the AlOx protection layer, and the sheet resistance did not change after the taping test as shown in Fig. 6(b).

**Figure 7:**
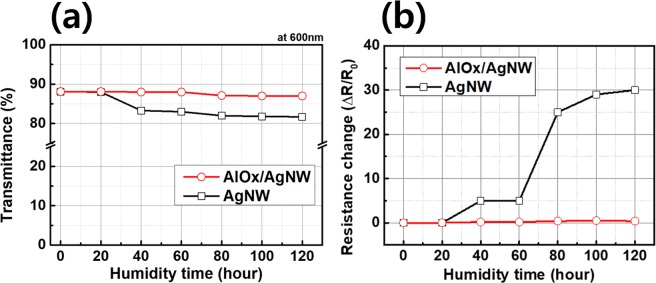
Transmittance (**a**) and Resistance change (**b**) of AgNWs with and without AlOx layer as a function of humidity time at 85 °C.

In order to confirm the effect of moisture and temperature, AgNWs with and without AlOx coating layer were exposed to high temperature and high humidity condition (85 °C, 85%) for 120 h after an outer bending test with radius of a 5 mm and 1000 cycles as shown in Fig. 7. Figure 7(a) shows the transmittance of AgNWs with and without the AlOx layer as a function of humidity time at 85 °C, the transmittance of AgNWs without the AlOx layer reduced to 79.8% after exposed for 120 h by oxidation of AgNWs. Especially, their transmittance dramatically decreased from exposure for 40 h. However, in the case of AgNWs with the 3 nm-thick AlOx protection layer, the transmittance slightly decreased from 88.1% to 87% after the humidity test at 85 °C for 120 h. Although the transmittance of AgNWs without the AlOx protection layer dramatically decreased after the humidity test at 85 °C, the transmittance of AgNWs with the AlOx protection layer did not decrease much after humidity and temperature test. Figure 7(b) shows the resistance change results of AgNWs with and without the AlOx layer after the humidity test at 85 °C. The resistance change of electrodes can be expressed as ΔR = (R − R0), where R and R0 represent the measured resistance and initial resistance, respectively. The resistance change of AgNWs without the AlOx layer was increased after the humidity test for 120 h. In contrast, AlOx covered AgNWs maintained their initial low resistance without any change after the humidity test at 85 °C. As expected from optical and electrical property results after humidity and temperature test, we can expect that oxidation of AgNWs leads to deteriorating the optical and electrical properties, and the AlOx layer was isolated well from external oxygen to AgNWs.

**Figure 8:**
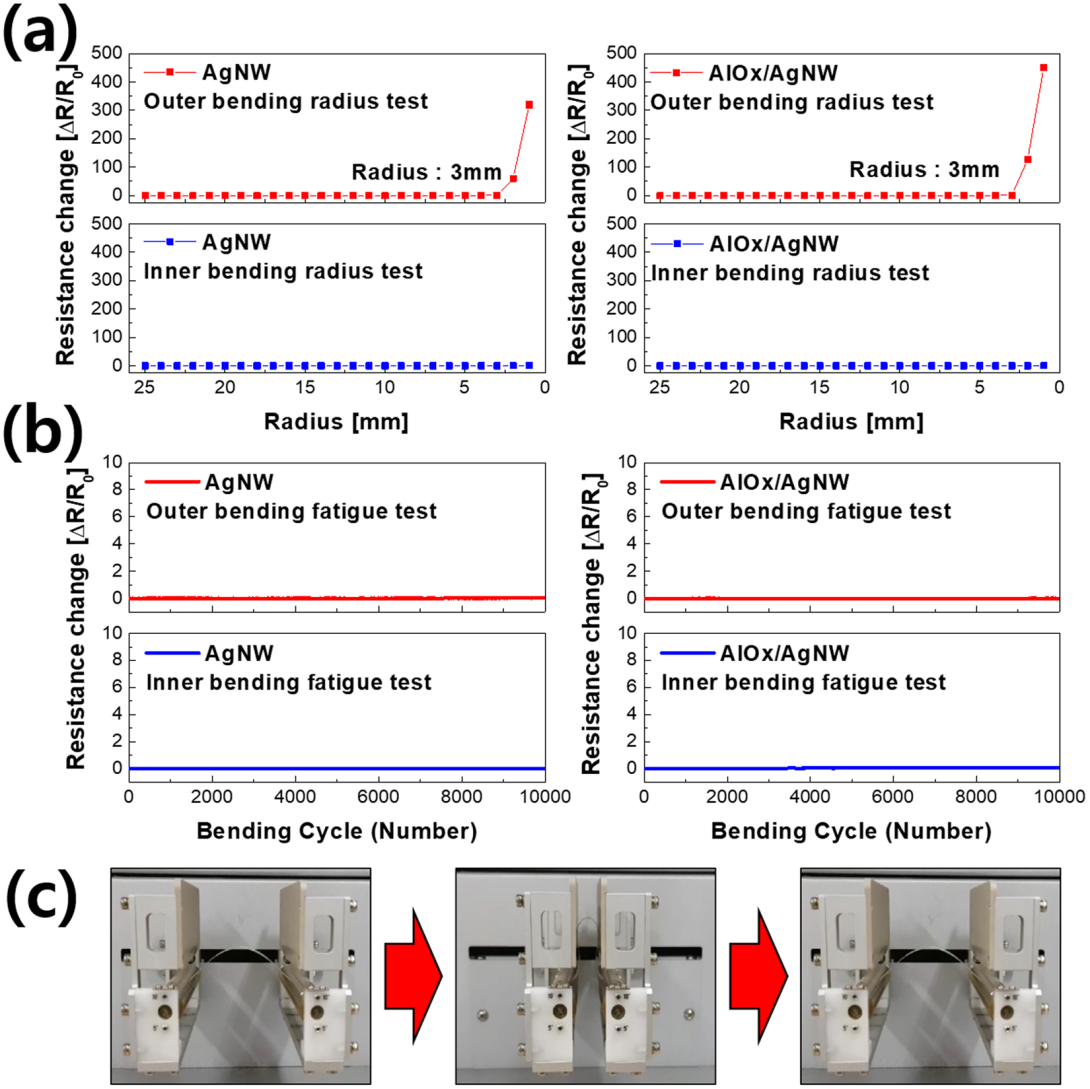
(**a**) Inner and outer bending test results of AgNWs with and without AlOx layer. (**b**) Inner and outer reliability bending test with and without AlOx layer. (**c**) Picture of cycling bending steps for the bending test.

Figure 8(a) shows the outer and inner bending test results of AgNWs with and without the AlOx layer. As a result, regardless of the AlOx layer, their resistance is increased the bending radius of less than 3 mm after the outer bending test. In addition, the resistance of AgNWs with and without the AlOx layer did not change until the inner bending radius of 1 mm. The outer and inner bending reliability test was also performed as shown in Fig. 8(b). AgNWs with and without the AlOx layer exhibited a constant resistance throughout the 10,000 bending cycles at a fixed bending radius of 3 mm. From these results, we confirm that AgNWs indicate superior mechanical flexibility regardless of the AlOx layer. Figure 8(c) shows cycling bending steps for the bending test.

**Figure 9:**
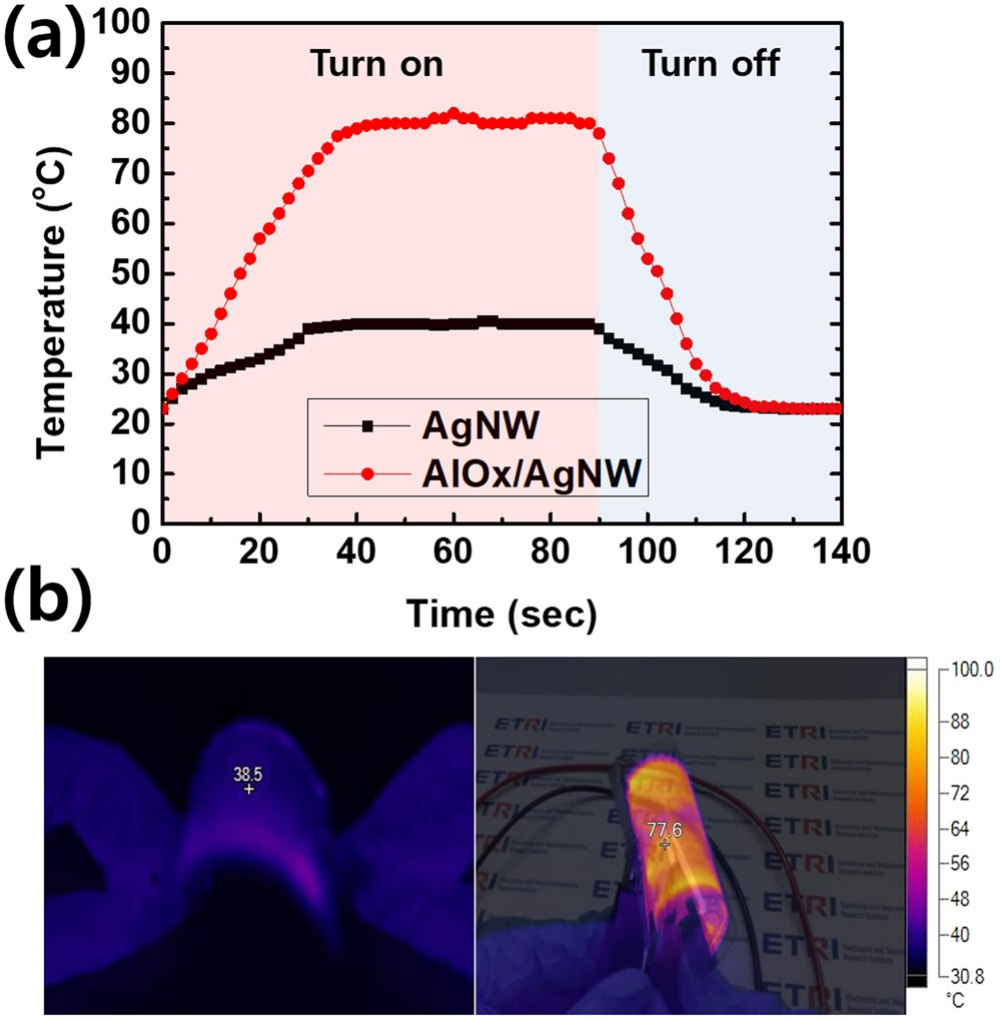
(**a**) Sheet temperature results of AgNWs with and without AlOx layer as a function of heating time when the input voltage of 7 V. (**b**) IR images of AgNWs without AlOx layer (left) and with AlOx layer (right) under different deformation modes.

Finally, we fabricated flexible transparent heaters (120 × 70 mm) using AgNWs with and without the AlOx layer. In the AgNWs without AlOx layer, the temperature increased only to 40 °C for 40 sec when the input DC voltage was 7 V with a joule heat generation of 0.21 W. However, In case of 3 nm-thick AlOx covered AgNWs, the temperature rapidly increased to 81 °C within 40 sec when the input DC voltage was 7 V with a joule heat generation of 0.77 W as shown in Fig. 9(a). Also, the temperature of AgNWs with and without AlOx decreased to room temperature within 40 sec after turn off the DC bias.

Figure 9(b) shows the IR images of AgNWs without the AlOx layer (left) and with the AlOx layer (right) under different deformation modes are taken by an infrared camera while the constant voltage of 7 V was supplied. The heaters using by AlOx covered AgNWs exhibits stable heating performance with uniform temperature distribution when bent or twisted.

**Figure 10:**
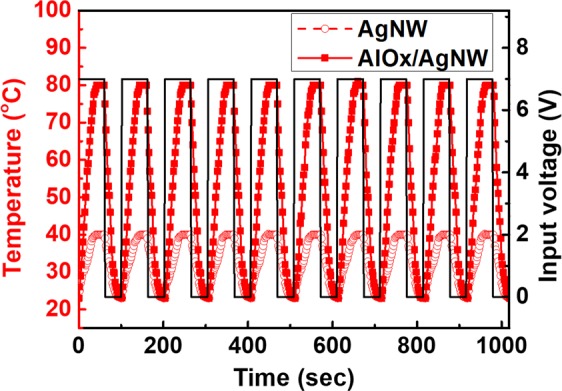
On/off responses of AgNWs based heater with and without AlOx layer.

Figure 10 shows the on/off response of the flexible heater using by AgNWs with and without the AlOx layer at DC voltage of 7 V. The cycling curve shows a relatively stable temperature recoverability of the flexible transparent heater. Therefore, this work is expected to be helpful for the development of high performance, reliable, and flexible transparent conductive film for the fabrication of uniform film heaters.

## Conclusion

In this study, we have demonstrated the effects of the AlOx protection layer by the reactive sputtering method on the electrical, optical, and structural properties of AgNWs prepared by drop-casting and air-dry process. The sheet resistance of the AlOx covered AgNWs decreased from 81 ohm/sq (without AlOx layer) to 47 ohm/sq (with 3 nm-thick AlOx layer) without transmittance loss. The significantly lower sheet resistance of AgNWs after deposited the AlOx layer can be attributed to increasing contact area at the junction of AgNWs. Furthermore, the strong networking of AgNWs by covered the AlOx layer lead to the improvement of AgNWs adhesion to the substrate. We indicate that since the AlOx layer protects AgNWs from external oxygen, the electrical and optical properties of AlOx covered AgNWs did not much change after the high humidity environment at 85 °C for 120 h. We fabricated a flexible transparent heater with AlOx covered AgNWs. The heater temperature increased to 81 °C within 40 sec at DC voltage of 7 V. Besides, Fast response and uniform temperature distribution have been achieved. These results strongly suggest that the AlOx covered AgNWs has the potential for the high stability flexible transparent heater applications.
